# Effects of Selenium Supplementation on Sperm Parameters and DNA-Fragmentation Rate in Patients with Chronic Autoimmune Thyroiditis

**DOI:** 10.3390/jcm10163755

**Published:** 2021-08-23

**Authors:** Rossella Cannarella, Rosita A. Condorelli, Aldo E. Calogero, Vincenzo Bagnara, Antonio Aversa, Emanuela A. Greco, Antonio Brunetti, Sandro La Vignera

**Affiliations:** 1 Department of Clinical and Experimental Medicine, University of Catania, 95123 Catania, Italy; rosita.condorelli@unict.it (R.A.C.); acaloger@unict.it (A.E.C.); sandrolavignera@unict.it (S.L.V.); 2 Paediatric Surgery Unit, Polyclinic G.B. Morgagni, 95123 Catania, Italy; vincenzobagnara@gmail.com; 3 Department of Experimental and Clinical Medicine, University Magna Graecia, 88100 Catanzaro, Italy; aversa@unicz.it; 4 Department of Movement, Human and Health Sciences, University of Rome “Foro Italico”, 00135 Rome, Italy; emanuela.greco@unicz.it; 5 Department of Health Sciences, University Magna Graecia, 88100 Catanzaro, Italy; brunetti@unicz.it

**Keywords:** selenium, sperm parameters, sperm DNA fragmentation, chronic autoimmune thyroiditis

## Abstract

Background: Selenium (Se) is an essential component of selenoenzymes, which have catalytic and antioxidant functions. A low Se status has been reported in patients with chronic autoimmune thyroiditis (AT) who benefit from Se supplementation. The role of Se in male reproduction is still a matter of debate. Although Se and selenoenzymes ensure sperm viability and protect against increased oxidative stress, only a few studies have assessed the effects of the administration of Se alone on sperm parameters, providing contrasting results. Aim: The aim of this study was to assess the effects of oral Se supplementation on conventional sperm parameters and DNA fragmentation (SDF) in patients with AT of reproductive age with normal thyroid function. Patients and Methods: Only patients with AT and normal thyroid function were selected for this study. All included patients underwent oral Se supplementation at the dose of 83 µg once daily (Syrel^®^, IBSA) for six months. Sperm conventional parameters, SDF, and thyroid function were assessed before and at the end of the treatment. Results: Twenty AT patients with normal weight were enrolled. After Se supplementation, they showed a higher sperm concentration, a higher percentage of sperm with progressive motility, and a higher percentage with normal morphology. They also had lower semen leukocyte concentration, and a lower percentage of spermatozoa with DNA fragmentation compared with pre-treatment values. Free-thyroxine serum levels increased significantly, whereas free triiodothyronine showed an upward trend. The thyroid-stimulating hormone did not change significantly. Conclusion: Se supplementation may represent a possible non-hormonal therapeutic choice for the treatment of male infertility, although further studies are needed to confirm this evidence. The possible thyroid hormone dependency of these findings needs to be clarified.

## 1. Introduction

Male infertility is widespread in industrialized countries, with an increasing prevalence estimated at 7% in 2011, and up to 12% in recent years [1]. Non-hormonal treatment of male infertility includes antibiotics, fibrinolytic, and antioxidants [2], the latter including nutrients and minerals.

Selenium (Se) is a micronutrient and a component of the so-called “selenoproteins”, or selenium-containing enzymes with a wide range of functions ranging from antioxidant and anti-inflammatory properties to the synthesis of thyroid hormones [3]. As for the latter, Se can enhance the enzymatic activity of various proteins involved in the synthesis of thyroid hormones, such as deiodinases. In particular, Se stimulates type 1 and 2 deiodinase to convert thyroxine into triiodothyronine by removing 5′-iodine, and also stimulates type 3, which inactivates triiodothyronine by 5′-deiodination [3].

Glutathione peroxidase (GPX) is a selenoprotein that degrades hydroperoxide excess into water, as well as organic hydroperoxides and phospholipid hydroperoxides, using glutathione as a co-substrate [4]. Particularly, GPX4 has been observed in sperm nuclei where it stabilizes condensed chromatin during sperm maturation [5]. Interestingly, Se seems to play a role in spermatogenesis, mainly acting in sperm maturation. Indeed, Gpx4-/- mice show low spermatogenic cells, sperm immobility due to altered mitochondrial function, and a hairpin-form tail [6,7,8]. Furthermore, selenoprotein 1 is a circulating protein reducing peroxynitrite and organic hydroperoxides [4]. This evidence supports the role of Se in the treatment of male infertility. However, to the best of our knowledge, the majority of the studies have focused on Se combined with other compounds, and only three randomized controlled trials (RCT) have investigated the effects of the administration of Se alone on sperm parameters, reporting contrasting results [9,10,11]. Therefore, more evidence is needed to confirm the effects of Se on sperm parameters.

Patients with chronic autoimmune thyroiditis (AT) may be more likely to have a low Se status because epidemiological data have shown that the prevalence of AT is higher in patients with a low Se status [3]. The latter may, in turn, predispose the patients to responsiveness to Se supplementation in terms of amelioration of sperm parameters. Furthermore, evidence supports the role of thyroid function (that can be influenced by Se supplementation [3]) in the modulation of sperm parameters. In fact, thyrotoxicosis can be associated with reduced seminal fluid volume, sperm concentration, motility, and morphology, whereas hypothyroidism is associated with a decreased percentage of spermatozoa with normal morphology [12].

On this basis, the present study was undertaken to evaluate the effects of Se supplementation on conventional sperm parameters and sperm DNA fragmentation (SDF) in patients with AT and abnormal sperm parameters.

## 2. Subjects and Methods

### 2.1. Patient Selection

This was a case–control study performed in patients with AT enrolled at the Unit of Endocrinology, Metabolic Diseases and Nutrition, University of Catania. Included patients were those of reproductive age, with normal thyroid function, and with at least one abnormal conventional sperm parameter (sperm concentration, total sperm count, motility, or morphology).

Diagnosis of AT was made based on the presence of serum antibodies against thyroid antigens (mainly to thyroperoxidase and thyroglobulin).

Patients with endocrine (pituitary, adrenal, dysfunction, hypovitaminosis D), metabolic (overweight, obesity, diabetes mellitus), and/or andrological (hypogonadism, varicocele, leukocytospermia, anti-sperm antibodies, male accessory gland infection/inflammation, history of cryptorchidism or urogenital surgery) diseases were ruled out.

All included patients underwent oral Se supplementation at the daily dose of 83 µg (Syrel^®^, IBSA Farmaceutici Italia S.r.l., Lodi, Italy) for six months. Sperm conventional parameters, SDF, and thyroid function were evaluated before and at the end of the treatment.

### 2.2. Sperm Analysis and Sperm DNA Fragmentation

Semen samples were collected by masturbation into a sterile container after 2–7 days of sexual abstinence and were analyzed immediately after liquefaction. According to the 2010 WHO guidelines, each sample was evaluated for seminal volume, pH, sperm count, progressive motility, morphology, and round cell concentration (WHO, 2010).

SDF was evaluated by flow cytometry using an EPICS XL (Becker Coulter, Milan, Italy). DNA fragmentation was evaluated by terminal deoxynucleotidyl transferase-mediated deoxyuridine triphosphate nick-end labeling (TUNEL) staining. The negative control was obtained by not adding terminal deoxynucleotidyl transferase to the reaction mix, while the positive control was obtained by pre-treating spermatozoa with 1 mg/mL of RNase-free deoxyribonuclease I (Sigma Chemical, St. Louis, MO, USA) at 37 °C for 60 min before labeling.

### 2.3. Hormone Measurements

Each patient underwent blood testing for the measurement of thyroid-stimulating hormone (TSH), free thyroxine (FT4), and free triiodothyronine (FT3) serum levels. The hormone evaluation was performed by electrochemiluminescence (Hitachi-Roche equipment, Cobas 6000, Roche Diagnostics, Indianapolis, IN, USA). The reference values were as follows: TSH 0.3–4.2 µIU/mL, FT4 9.3–17 ng/L, and FT3 2–4.4 pg/mL.

### 2.4. Statistical Analysis

Results are reported as mean ± SD throughout the study. The data distribution was analyzed by the Shapiro–Wilk test. Mean differences were evaluated by the Student’s t-test or Wilcoxon test for paired samples in normally or non-normally distributed variables, respectively. A chi-squared test was used to evaluate differences in the prevalence of abnormal sperm parameters before and after the treatment. Significance was accepted for a *p*-value <0.05. Statistical analysis was performed using MedCalc Software Ltd. (Version 19.6—64 bit, https://www.medcalc.org (accessed on 6 July 2021)).

### 2.5. Ethical Approval

This study was conducted at the Division of Endocrinology, Metabolic Diseases, and Nutrition of the teaching hospital “G. Rodolico—San Marco”, University of Catania (Catania, Italy). The protocol was approved by the internal Institutional Review Board and informed written consent was obtained from each participant after a complete explanation of the purpose and nature of all procedures used. The study was carried out according to the principles of the Declaration of Helsinki.

## 3. Results

**Figure 1:**
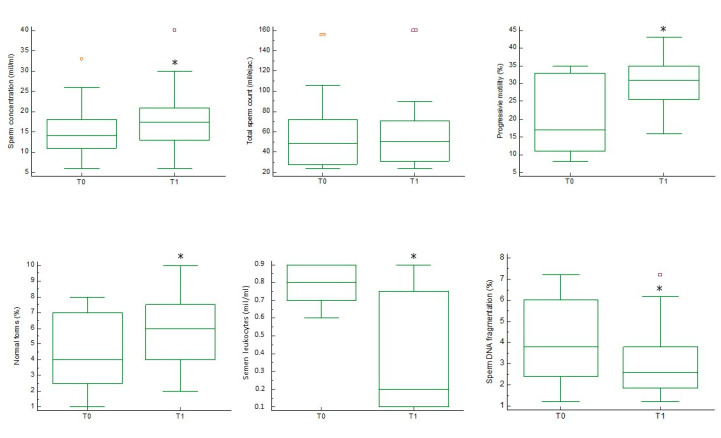
Conventional sperm parameters before and after selenium administration. Sperm concentration, total sperm count, progressive motility, morphology, leukocytes, and sperm DNA fragmentation are shown before (T0) and after (T1) selenium administration. * *p* < 0.05 vs. T0 (Student’s *t*-test). Orange and purple symbols represent the maximum values.

Twenty patients (age 32.2 ± 7.1 years) with normal body mass index (23.0 ± 1.5 Kg/m^2^) were enrolled. After supplementation with Se, patients showed higher sperm concentration (15.7 ± 6.7 vs. 18.0 ± 8.2, *p* < 0.05), higher percentage of sperm with progressive motility (19.8 ± 10.3 vs. 30.4 ± 6.8, *p* < 0.0001), and higher percentage with normal morphology (4.5 ± 2.4 vs. 5.8 ± 2.0, *p* < 0.005). They also showed a lower concentration of leukocytes in the seminal fluid (0.8 ± 0.1 vs. 0.4 ± 0.3, *p* < 0.0005), and a lower percentage of spermatozoa with DNA fragmentation (3.9 ± 0.2 vs. 2.9 ± 1.6, *p* < 0.01) compared to pre-treatment values. No significant difference was observed in total sperm count (59.1 ± 40.0 vs. 61.2 ± 38.9) (Figure 1).

The prevalence of teratozoospermia was significantly reduced after treatment compared to pre-treatment values (45% vs. 10%, *p* < 0.05). Se supplementation did not significantly change the prevalence of oligozoospermia (50% vs. 35%), asthenozoospermia (70% vs. 70%), and spermatozoa with DNA fragmentation (30% vs. 10%).

**Figure 2:**
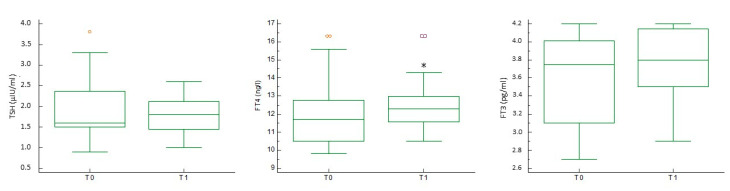
Thyroid function before and after selenium administration. The serum concentrations of thyroid-stimulating hormone (TSH), free-thyroxin (FT4), and free triiodothyronine (FT3) are shown before (T0) and after (T1) selenium administration. * *p* < 0.05 vs. T0 (Student’s *t*-test). Orange and purple symbols represent the maximum values.

Regarding thyroid function, serum FT4 levels increased significantly (12.0 ± 2.1 vs. 12.5 ± 1.6, *p* < 0.05), whereas FT3 showed an upward trend (3.6 ± 0.5 vs. 3.8 ± 0.4, *p* = 0.05). TSH did not change significantly (1.9 ± 0.7 vs. 1.8 ± 0.5) (Figure 2).

Finally, patients did not experience any of the side effects of excessive Se administration, such as diarrhea, fingernail weakening, garlic odor in breath and sweat, hair loss, irritability, itchy skin, nausea and vomiting, unusual tiredness, and weakness.

## 4. Discussion

With the increase in the prevalence of male infertility, the possible benefits of nutraceutical administration on improving conventional and biofunctional sperm parameters, as well as the pregnancy rate, have been questioned. As an essential component of selenoenzymes—proteins with catalytic and antioxidant functions—is Se, which has been considered as a promising treatment to be used to improve sperm parameters. In this context, the present study was undertaken to evaluate the impact of oral administration of Se for six months on conventional sperm parameters and SDF. In a highly-selected population with AT, we found that Se supplementation can improve conventional sperm parameters and SDF, and increase FT4, thus suggesting that the oral administration of Se for six months could be considered useful for the treatment of male infertility.

Previous studies have already attempted to evaluate the role of Se administration on sperm quality. In particular, two meta-analyses have been published so far on this topic. The first included seven randomized, double-blind, placebo-controlled studies on the administration of Se, carnitine, and the co-enzyme Q10. Among the included studies, only two administered Se alone (200 µg/day and 100 µg/day, for 26 and 12 weeks, respectively) [10,11]. The sub-analysis of these studies showed the greater efficacy of Se compared to placebo, as Se significantly improved sperm concentration and motility [13]. The other meta-analysis included 15 articles in the quantitative synthesis and reported an improvement in sperm concentration, total count, and normal morphology following Se supplementation [14]. However, the vast majority of included studies administered Se in combination with other compounds. Only one of them [9] examined the effects of administering Se alone (300 µg/day for 48 weeks), reporting no significant effects. The authors commented that the lack of efficacy may reflect the fact that testicular Se reserves were not affected, so no improvement can be seen through the tripling of dietary Se intake [9]. This consideration underlines the importance of the choice of the population to be treated. In the present study, patients with AT, who according to the available data are at risk for low Se status [3], benefited from the administration of Se.

This study also proved the efficacy of Se supplementation on SDF, at least in the selected population. To the best of our knowledge, no study has investigated this parameter in vivo. A recent in vitro analysis reported that the incubation of sperm samples from asthenozoospermic patients with Se 2 μg/mL for up to 6 h resulted in an improvement of sperm motility, viability, mitochondrial potential, and SDF [15]. In addition, the concentrations of malondialdehyde also decreased [15], thus supporting the antioxidant properties of Se. This finding may be explained by the improved activity of the sperm-specific GPX4, due to the higher Se availability. GPX4, in turn, would counteract oxidative stress and hinder DNA damage [5]. To the best of our knowledge, this is the first study documenting an efficacy on sperm quality with a low dose of Se supplementation. Accordingly, in the studies considering the sperm conventional parameters as the primary outcome, the range of Se administered is from 100 to 300 µg daily [13]. In contrast, in those studies considering thyroid function as the primary outcome, the range is 83–200 µg daily [3]. Our study design considers sperm parameters as the primary outcome, in a cohort of patients with AT at risk for a low Se status. This is the first evidence reporting the improvement of conventional sperm parameters and SDF with a dose of 83 µg.

In the present study, the administration of Se resulted in a slight increase in serum FT4 levels. FT4 appears to have a positive impact on sperm quality [16]. The in vitro incubation of spermatozoa with FT4 has in fact increased sperm progressive motility, and has improved the sperm mitochondrial function, the compactness of sperm chromatin, and SDF at a concentration of 2.9 pmol/l, which is close to the physiological FT4 concentrations in the seminal fluid of euthyroid men [16]. The influence of thyroid function on sperm quality has already been reviewed [12]. Thyroid hormones can affect the function of several cells, such as Sertoli, Leydig, and germ cells. Serum thyroid hormone levels outside the normal range alter conventional sperm parameters and, therefore, the evaluation of thyroid function should be part of the diagnostic process of male infertility [12]. Despite this evidence, we cannot claim that the slight increase in FT4 levels contributed to the improvement in sperm parameters found in this study. However, their influence cannot be completely ruled out.

This study is limited by the low sample size (which, however, is similar to those of previous studies (e.g., [11])), the lack of a control arm, and the absence of data on the patients’ Se status before and after supplementation. On the other hand, the highly selected population and the long-term Se supplementation (6 months) represent the strengths of the present study.

## 5. Conclusions

In conclusion, this study provides evidence in favor of the effects of Se administration on conventional sperm parameters. Only three studies have evaluated this specific issue, and conflicting data have been reported. Furthermore, this is the first time that the effects of Se supplementation on SDF have been evaluated through an in vivo study design. Overall, our data support the benefits of oral Se administration on SDF, especially when a high-risk population for low Se status is selected.
